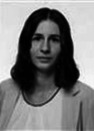# Hydroxymethylfurfural and its Derivatives: Potential Key Reactants in Adhesives

**DOI:** 10.1002/cssc.202001539

**Published:** 2020-08-28

**Authors:** Catherine Thoma, Johannes Konnerth, Wilfried Sailer‐Kronlachner, Thomas Rosenau, Antje Potthast, Pia Solt, Hendrikus W. G. van Herwijnen

**Affiliations:** ^1^ Wood K plus – Competence Center of Wood Composites and Wood Chemistry Kompetenzzentrum Holz GmbH Altenberger Str. 69 4040 Linz Konrad-Lorenz Str. 24 3430 Tulln Austria; ^2^ Department of Material Science and Process Engineering Institute of Wood Technology and Renewable Materials BOKU University of Natural Resources and Life Sciences-Vienna Konrad-Lorenz Str. 24 3430 Tulln Austria; ^3^ Department of Chemistry, Institute of Chemistry of Renewable Resources BOKU University of Natural Resources and Life Sciences Muthgasse 18 1190 Vienna Austria

**Keywords:** adhesives, biomass valorization, industrial chemistry, renewable resources, sustainability

## Abstract

5‐Hydroxymethylfurfural (HMF) is a promising bio‐derived platform chemical with a broad scope of application, for example, in the production of solvents, fuels, polymers, or adhesives. The wood and foundry industries are among the largest adhesive users and currently both rely to a large extent on the use of fossil‐based binders, such as by using formaldehyde as a crosslinker in many commercial adhesive systems. The industry is thus looking for suitable alternatives to replace fossil‐based chemicals. HMF and its derivatives are considered to be key renewable reactants in adhesive systems. The core of this Review is the critical evaluation of the potential of HMF and its derivatives in adhesive systems. The technological performance was assessed in the fields of wood‐based materials, sand casting and composites. As an overall conclusion, HMF and its derivatives have a high application potential in alternative adhesives. Clearly, further research is needed to improve the performance and produce economically competitive adhesives.

## Graphical abstract

**Sticks like glue**: This Review critically assesses the role of 5‐hydroxymethylfurfural (HMF) and its derivatives as renewable key reactants in adhesives. Application fields range from wood‐based materials, sand casting, and composites. The technological performance of many HMF‐based adhesives reaches the minimum requirements, but further research on economically feasible HMF‐based resins is needed.

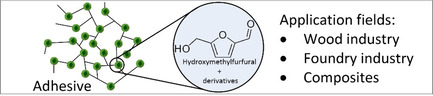

## 1 Introduction

For a sustainable development towards bioeconomies, the use of renewable resources must be enhanced and more sustainable ways for material production must come into focus. Bio‐derived 5‐hydroxymethylfurfural (HMF) is considered a high‐value platform chemical and an important intermediate for derivatives, such as 2,5‐furandicarboxylic acid (FDCA), which has been listed as one of the top value‐added chemical by the US Department of Energy in 2004.^1^ HMF and its derivatives are promising key reactants among others in the adhesive production.

In general, adhesives are nonmetallic substances that are capable of joining materials by surface bonding (adhesion) with bonds possessing adequate internal strength (cohesion).^2^ Most commercial adhesives, such as phenol–formaldehyde (PF), phenol–urea–formaldehyde (PUF), urea–formaldehyde (UF) or melamine–formaldehyde (MF), are still fossil‐based. The decreasing availability of fossil resources in the long run and rising environmental concerns related to emissions of volatile organic compounds promote the use of renewable resources in material production. Today, the main crosslinker used in the aforementioned adhesive systems is formaldehyde, which is classified as Carcinogen Category 1B according to the regulation of the European Parliament and the European Council 605/2014. Moreover, increasing regulations and public concern about emission of hazardous substances and volatile organic compounds from chemical binders bring the use of alternative adhesives to the forefront. In 2011, the total annual worldwide production capacity of formaldehyde was 18×10^6^ t (for 100 wt% formaldehyde) with an annual global consumption of 13.1×10^6^ t.^3^ In 2017, the production of urea‐, phenol‐, and melamine‐formaldehyde resins (UF, PF, MF) consumed almost 70 % of the global formaldehyde production.^4^

In general, the wood product manufacturers are the largest adhesive user. Wood adhesives make up more than 65 % by volume of all the adhesives used worldwide.^5^ Other large application fields include insulation materials and foundry materials. In order to highlight the importance of the development of alternative binders in the aforementioned application fields, one has to look at the production capacity and amount of adhesive used in these industries. In particleboard production, mainly formaldehyde‐based condensation binders, such as UF or MUF, are used. In general, for the production of wood‐based materials such as particleboard, rather large amounts of binder (10–14 %) relative to dry wood are needed.^6^ In 2017, 95.3 million cubic meters of particleboard were produced worldwide, at an average density of 650 kg m^−3^ the amount of binder used annually adds up to 6.20×10^7^ t (at 10 % binder content).^7^

A typical application field of glass fiber (mineral wool)‐PF composites are insulating materials.^8^ For the insulation of buildings often batts and rolls are used that have a typical density of 8–16 kg m^−3^ and a binder content of 3–7 wt%,^9^ which is lower compared to the application in the wood panel industry. In 2007, the overall mineral wool production was 40 million m^3^ per year, which translates into about 400 000 t per year of resin.^10^ In 2007, the global metal casting industry produced 94.9 million metric tons of metal parts, with typically 1–2 % (based on sand and depending on application) binder being used in the cold box process.^10^

For a technical suitability, the used crosslinkers must meet various criteria from a chemical and application perspective. Previous reviews providing an overview of alternative adhesive systems for the wood^11^ and foundry^12^ industry mainly focus on the comparison of various renewable‐based adhesives for wood‐based panels, such as lignin, tannin, protein, or carbohydrate‐based adhesives, but the potential of HMF in alternative adhesive systems has not yet been thoroughly evaluated. A review by Solt et al.11a included examples of previously reported HMF‐based adhesive systems but did not further elaborate the potential of HMF and its derivatives. Kaczmarska et al.^12^ reviewed the application of polysaccharides in the foundry industry but did not include HMF.

Many of the reported HMF‐based and HMF derivative‐based adhesives were already produced with a specific application in mind. The majority of it being wood‐based materials, such as particleboard, followed by the application as a polymer matrix in composites, for example, in insulation materials.

A thorough study was done on previous reports on HMF‐based adhesives formed in situ from carbohydrates. Another approach reported in literature was the utilization of HMF as modifiers or substituent in conventional adhesives. Adhesives based on HMF derivatives were included as well. For this Review, scientific publications as well as patent applications were considered. In many early publications the formation of HMF was only speculated, but for a holistic picture these publications were included as well.

In general, the focus of reported studies was either on the production of HMF‐based adhesives or on the manufacture of products in which the HMF‐based adhesive is only one component. Structure‐property relations provide insight needed for the development and optimization of products using the adhesive bonding technique. Consequently, this Review assesses the chemical structure and composition of the reported adhesives and discusses the material properties of the designed products with regard to the requirements of the specific application

The aim of this Review is to identify the potential of HMF and its derivatives as key reactants in adhesive systems by summarizing reported accounts. It provides an overview of current HMF‐based adhesives and potential application fields. The influence of HMF on the material properties is discussed, especially with regard to the requirements of the specific applications.

## 2 HMF as Sustainable Key Reactant

**Scheme 1:**
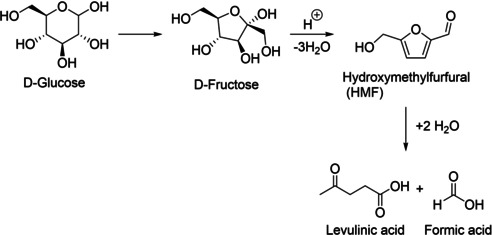
Acid‐catalyzed dehydration of monosaccharides to HMF and rehydration to give levulinic acid and formic acid.

The acid‐catalyzed dehydration of hexoses, such as glucose or fructose, leads to HMF. This mostly thermally activated process involves cyclization to the five‐membered heterocyclic furan ring and elimination of three equivalents of water (Scheme 1). HMF is characterized by the weekly aromatic furan system, an aldehyde function and a hydroxymethyl group, which is comparable to a benzyl alcohol moiety at the benzene system. A review on the chemistry of HMF, process chemistry and technologies was published by van Putten^13^ in 2013. Considerable efforts are being made to produce HMF in larger quantities. Since 2014, a small‐scale, commercial production plant with a capacity of 300 t per year of HMF is operative.^14^ The large‐scale production of HMF is still facing some chemical and technological challenges, an important chemical one being its conversion into levulinic acid and formic acid in aqueous mixtures under uptake of two equivalents of water, in an acid‐catalyzed rehydration‐fragmentation sequence (Scheme 1).

Pizzi^5^ defined three main ways for the utilization of carbohydrates in wood adhesives:

- Binder production from carbohydrates by forming degradation compounds (e. g., HMF)
- Modification of existing adhesives (e. g., PF and UF)
- Direct usage as wood adhesives

Based on this, the integration of HMF and its derivatives in binder systems can be classified in three approaches. The first one is the in situ conversion of carbohydrates into a reactive intermediate (e. g., HMF or HMF derivatives), which then directly forms a high molar mass network with other monomers. Such an in situ approach avoids the challenges of producing and isolating HMF in high yield and reduces the risk of rehydration of HMF to side‐products, such as levulinic acid and formic acid.

**Scheme 2:**
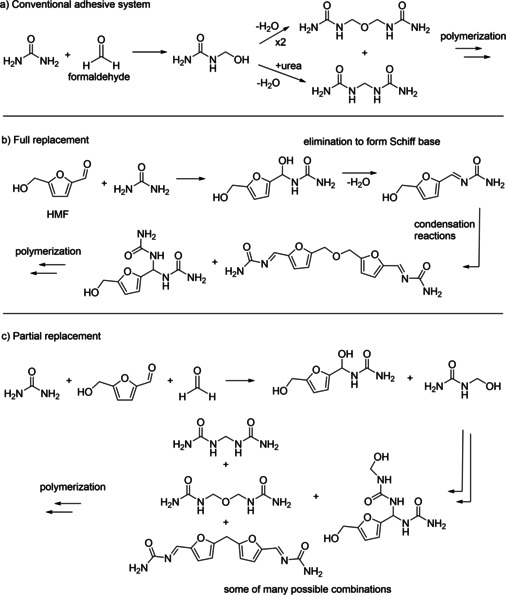
Replacement of formaldehyde as crosslinker with HMF as alternative crosslinker: a) Conventional adhesive system (e. g., UF‐resin); b) full replacement of formaldehyde with HMF; c) partial replacement of formaldehyde with HMF. Only monomethylolation of urea is depicted, although formation of higher methylolated derivatives takes place as well.

The second approach is the incorporation of HMF as an alternative key reactant into an existing adhesive system formulation, such as the (partial) substitution of formaldehyde (Scheme 2). The main advantage of this approach is that the reaction mechanism and properties remain in an ideal case quite similar to the traditional adhesive. As can be seen in Scheme 2, full replacement and a partial replacement of the crosslinker can be distinguished. In the latter case, HMF is just a modifier. In Scheme 2, only exemplary reactions are given; for examples the reaction between HMF and formaldehyde and that between urea and formaldehyde are depicted, whereas HMF could also form dimers or react with formaldehyde. Scheme 2 is meant to show the main reaction products rather than to fully illustrate the complex nature of these reaction systems.

The utilization of pure HMF as adhesive, which corresponds to the third of Pizzi's categories,^5^ has not been reported in the literature.

## 3 HMF‐Based Adhesives from In‐Situ Carbohydrate Conversion

In order to produce HMF as furanoid crosslinker in the adhesive systems, the carbohydrate feedstock needs to be converted first into suitable precursors which then have to be dehydrated. Several publications^15^ on binder systems that use carbohydrates under acid conditions hypothesize about the formation of intermediate HMF as a crosslinker.

The use of disaccharides (e. g., saccharose, lactose) and polysaccharides (starch) as carbohydrate feedstock for the production of resin systems for wood applications is already known.^16^ Polysaccharides have been extensively tested as wood adhesives due to their high molecular weight and the availability of polar and hydrogen‐bonding functional groups, which exhibit good adhesion to high surface energy adherends, such as wood or metal. An extensive review on utilization of polysaccharides in adhesive systems was published by Patel et al.^16^

Disaccharides, oligosaccharides and polysaccharides are formed by two monosaccharides that are joined by a glycosidic bond. The acid used in the carbohydrate‐based adhesive formulations cleaves these glycosidic bonds and dehydrates the formed monosaccharides to HMF, which in turn polymerizes. The main saccharides used for resin systems are glucose, fructose, galactose as monosaccharides, sucrose and lactose in case of disaccharides and starch as polysaccharide. The unreacted carbohydrates are integrated into the polymer network as additional crosslinkers, owing to their hydroxy (and sometimes carboxylate) groups.

### 3.1 Wood adhesives

Early work on carbohydrate‐based binder systems was done with phenol as the monomer to produce thermosetting resins for wood‐based materials, such as plywood or particleboard. Meigs^17^ patented one of the earliest approaches for an in situ production of a dehydrated carbohydrate‐phenol resin in 1926. HMF was proposed to act as the crosslinker, but no structural or mechanical analysis of the produced resins was performed, although water elimination of the produced resins was analyzed. Most early studies hypothesized about the formation and involvement of HMF in the crosslinking of the compounds. Since no proof of HMF formation was provided, the accuracy of the proposed mechanism remained an open question.

The same holds true for a patent from 1976 of Chemical Process Corporation CPC15d describing a process in which phenol reacted with hexoses in the presence of a Lewis acid catalyst. The postulated reaction mechanism included the acid dehydration of glucose to form HMF, which then formed condensates with phenol.

Koch et al.15a, 15b, 15c studied the incorporation of starch and its hydrolysis products in phenolic resins. The starch was dehydrated to HMF using an acid catalyst in the presence of phenol and formaldehyde. They concluded that due to the formation of HMF/phenol condensates, 60 wt% of phenol and 50 wt% of formaldehyde could be saved in comparison to commercial phenol‐formaldehyde resins. The in situ hydrolysis reduced the risk for side reactions, such as the polymerization of HMF to humins or its decomposition to levulinic acid. In addition, the incorporation of HMF reduced the free formaldehyde content by a factor of 2–3 at 20 % formaldehyde replacement. At that point, formaldehyde emission from particleboard was already a rather hot topic and significant efforts were made to reach lower formaldehyde emission values. The explicit mentioning of HMF as replacement for formaldehyde with the potential to reduce formaldehyde emissions shows that the consideration of HMF as renewable substitute is not a new concept, but one that had already been of interest 40 years ago.

In 1985, Viswanathan et al.^18^ produced particleboards with resins obtained from the reaction of whey permeate, ammonium nitrate (NH_4_NO_3_), phenol and urea. Additional specimens obtained from pure HMF and urea, or phenol and ammonium nitrate, were produced and compared. It was found that HMF was formed in the dehydration of lactose and reacted with monomers present (urea, phenol). The authors hypothesized that the reaction of HMF with urea or phenol leads to short chain polymers, which are soluble in methanol, whereas in the presence of lactose a complex polymer can be formed, to which HMF contributes only a small fraction. The lack of information about the structure of these resins and the missing data on the mechanical performance are limitations, however, it is noteworthy that a number of authors have recognized the potential involvement of HMF in their developed resin formulations.

A patent assigned to Stofko^19^ describes a method of dehydration of monosaccharides to HMF and subsequent condensation with lignin and other phenolic compounds of wood. Unlike previous researchers, who employed Brønsted acids, Stofko used the Lewis acid aluminum chloride as a catalyst, along with ethylene glycol to adjust the viscosity if necessary. The highest internal bond (IB) strength was 1.25 N mm^−2^ at 190 °C press temperature and a press factor of 20 s mm^−1^. The internal bond strength (IB) is an important quality parameter for particleboard, more specifically of the adhesion of the core layer. It is the tensile strength perpendicular to the plane of the board and used for the classification of particleboards according to EU and US standards. The IB values must be used with caution if no standard testing procedure is used, since the density of the core layer, the particle properties and the geometry as well as the adhesive quantity and distribution may all influence the internal bond strength. The press factor refers to the curing speed, typically expressed as the time needed to cure 1 mm of board thickness.

In this particleboard an adhesive system of 18.6 % sucrose, 0.4 % aluminum chloride, 1.62 % ethylene glycol and 2.36 % ammonium nitrate was used (Table 2). The direct application of an acid to the wood surface and subsequent thermal treatment leads to hydrolysis of the polysaccharidic wood components. This hydrolytic degradation of hemicelluloses and cellulose was not limited to the wood surface but also affected adjacent layers, which reduced the strength significantly. This remote hydrolytic effect indicates some production of HCl with its low diffusivity by hydrolysis of the aluminum chloride (AlCl_3_) catalyst.

Several adhesive systems based on tannins and HMF have been described previously. Tondi et al.,^20^ for instance, investigated the gluing properties of starch‐saccharose formulations for particleboard production. Tannins have been used as a potential replacement for phenolic substances in adhesive systems. The authors hypothesized that at temperatures above 160 °C “caramelization” of saccharose takes place and furanoid compounds, such as HMF, 2‐hydroxyacetylfuran (HAF), and mono‐ and dihydroxydimethylfuranone (DDF) are produced. Those molecules are likely to be involved in the crosslinking of amylose and amylopectin (starch) and in the hardening reaction. The formulation of 53 % starch, 13 % sugar, and 33 % tannin gave the highest IB value of 0.40 N mm^−2^. The high process temperatures applied are unproblematic for inorganic insulation materials, but unsuitable in the manufacturing of wood panel boards where typical temperatures are in the range of 110–120 °C.

**Scheme 3:**
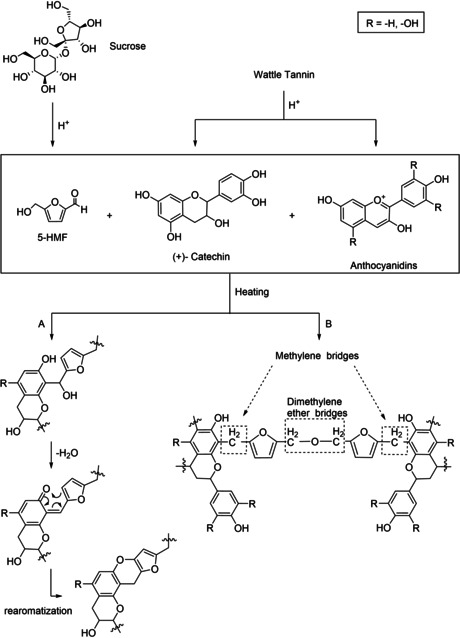
Reaction of sucrose/tannin/acid adhesives *via ortho*‐quinone methide (Path A) and reaction as proposed by Zhao et al. (Path B).^23^, ^24^

Zhao et al.^21^ investigated the incorporation of HMF derived from sucrose in various adhesive systems for particleboard manufacturing. The proposed adhesive systems were based on citric acid/sucrose, tannin/sucrose, tannin/sucrose/citric acid, tannin/sucrose/sulfuric acid. The authors assumed that sucrose was dehydrated to HMF, which then reacted with the tannin (Scheme 3). The FTIR spectra indicated the formation of HMF and the linkage with tannin by methylene bridges and methylene ether bridges. However, structural analysis was limited to FTIR and the detailed reaction mechanism was not investigated.

In a follow‐up study, Zhao et al.^22^ investigated the effect of citric acid in a tannin/sucrose adhesive in more detail. They proposed that citric acid accelerated the decomposition to HMF. The addition of citric acid reduced the required reaction temperature and decreased the required hot‐pressing temperature to 200 °C. The best results were obtained at a molar ratio of tannin: sucrose: citric acid=1 : 3 : 2. The bending properties [modulus of rupture (MOR)=21.5 N mm^−2^, modulus of elasticity (MOE)=4900 N mm^−2^] and the water resistance of the PB (thickness swelling TS=5 % after pressing at 220 °C) were increased. While it can be reasonably assumed that the addition of an acid has a beneficial catalytic effect with regard to dehydration and HMF formation, the other observed effects can rather be explained by the crosslinking ability of citric acid itself and its thermal degradation product itaconic acid, which leads to ester‐type, aldol type and Michael‐type condensation products. Moreover, the increased water resistance, which would be hard to explain solely based on high concentrations of nonreacted, highly hydrophilic citric acid, points to this conclusion. In another follow‐up study Sun et al.^23^ compared the effect of citric acid and hydrochloric acid on the mechanical properties of the particleboard. The high acidity of hydrochloric acid degrades the polymeric cellulose and hemicellulose, leading to inferior mechanical properties. In a comparative approach, sulfuric acid was used as a catalyst to improve the curing process of tannin/sucrose adhesives. Thermal analysis, insoluble mass proportion, FT‐IR and solid state ^13^C NMR spectroscopy were used to investigate the curing behavior. The results showed that HMF participated in the curing and formed methylene bridges with the C8 position of the resorcinol A‐rings of tannin and dimethylene ether bridges as chemical chain of the polymer (Scheme 3).

Many more alternative reactions are conceivable but were not reported. The risk of decomposition of cellulose and hemicellulose is also given for the utilization of sulfuric acid as catalyst. Also, the linkage of the furan system to two methylene groups at the same time is remarkable because the aldehyde function, upon condensation, should form a CH(OH) structure, which later on would give rise to *ortho*‐quinone methide formation and more highly condensed structures.

In general, the risk of hydrolysis of wood components and of corrosion in the processing line due to the low pH needed for these adhesive systems is a disadvantage in the manufacturing process of such wood‐based materials.

To overcome this, Zhao et al.^25^ developed an adhesive system based on the application of an ammonium dihydrogen phosphate catalyst for sucrose conversion reactions. They propose several reaction paths: first, sucrose is catalytically hydrolyzed to glucose and fructose. Then, fructose is dehydrated to HMF, and the catalyst is decomposed to phosphoric acid and ammonia. The ammonia would react with both glucose and HMF, and the authors assume that Amadori rearrangements and conversions of HMF into Schiff bases are proceeding as well. This seems somewhat unlikely since at the prevailing high acidity NH_3_ should not be present as the free base, but only as ammonium cation, which is non‐nucleophilic and would hardly undergo such processes. At a press temperature of 200 °C they reached an IB value of IB=1.4 N mm^−2^, MOR=24.5 N mm^−2^, MOE=5200 N mm^−2^, and TS=9.2 %. The produced particleboards fulfilled the requirements of JIS A 5908 standard, but the hot pressing time and resin content were relatively high (press factor 67 s mm^−1^, 20 wt%) and not suitable for an economic industrial production.

Knauf Insulation SPRL has filed several patents^26^ on binders made out of citric acid, glucose and a nitrogen source for wood products. In examples given in the patent, the IB strength was approximately 0.4 N mm^−2^ at a rather high press factor (time needed to cure 1 mm^2^ of particleboard) of 50 s mm^−1^ and a press temperature of 220 °C. The density of the particleboards was in the range of 550–640 kg m^−3^ and the amount of resin was 12–14 % (weight of resin solids to weight of dry wood). Mechanistic insights were not provided. A comparison of the mechanical properties of HMF‐based wood adhesives can be found in Table 2.

### 3.2 HMF–glass fiber composites

For the production of composites based on glass fiber, higher temperatures (180–250 °C) are applied for the curing of the resins. In addition, a more acidic binder can be used since glass fibers are not degraded in contrast to the polysaccharidic wood components. The strength needed for the composite materials varies depending on the intended application.

In a recent publication, Yuan and Zhang^27^ produced a phenol/HMF resin (PHMF) in an in situ process from glucose using a chromium chloride, CrCl_2_/CrCl_3_, and tetraethylammonium chloride (TEAC) as catalysts. The reaction was performed at 120 °C for 8 h leading to a glucose conversion of more than 90 % with concentrations of free HMF below 1.5 wt%. Glass fiber‐PHMF composite specimen were produced and compared to glass fiber‐PF composite counterparts. This production method was also patented by the University of Western Ontario.^28^ Zhang et al.^29^ tested various curing agents, organosolv lignin (OL), Kraft lignin (KL), hexamethylene tetramine (HMTA) in continuation of their previous work. The fiber glass‐PHMF samples were cured at temperatures ranging from 120–180 °C at curing times of 30 and 60 min. The mechanical properties of the PHMF containing composites were significantly lower when OL or KL was used for curing of the PHMF resin. Zhang et al.^30^ also studied the curing with a bisphenol A type epoxy resin. In this study, the formaldehyde‐free curing agent bisphenol A diglycidyl ether (DGEBA) was used to crosslink the PHMF resin. The mechanical properties of this resin were comparable to the ones cured with HMTA.

The reference tensile shear strength for long glass fiber reinforced thermosets made out of PF is 115 N mm^−2^ (Table 1). The tensile shear strength of the glass fiber‐PHMF composites cured with HMTA showed satisfactory results (Table 1). In addition to their work on PHMF, Zhang et al.^31^ studied a bio‐phenol‐HMF resin (BPHMF). Hydrolysis lignin was degraded to de‐polymerized hydrolysis lignin (DHL) to partially substitute phenol. Glucose was dehydrated to HMF, which then reacted with phenolated DHL in an in situ process, giving a yield of 85 %. However, the tensile strength of the produced BPHMF‐glass fiber composites was significantly lower compared to the PHMF‐glass fiber composites produced with phenol. The research of Zhang et al. provided a comprehensive analysis of PHMF resins for composites. It is noteworthy that a full replacement of formaldehyde with HMF was possible and the utilized HMF was directly synthesized from glucose. However, in the light of increasing environmental awareness and green chemistry principles, the use of toxic chromium salts and ecotoxic quaternary ammonium salts is rather questionable and clearly leaves room for further improvement.

**Table 1:** Mechanical properties of glass fiber‐phenol‐HMF composites.

Material	Tensile strength [N mm^−2^]	Ref.
Long glass fibers reinforced thermoset (PF)	115	[10]
Glass fiber‐PHMF composite (HMTA)	127±2	[27]
Glass fiber‐PF composite (HMTA)	118±1	[27]
PHMF+OL	99±5	[29]
PHMF+KL	93±2	[29]
PHMF+HMTA	109±4	[29]
PHMF+DGEBA	105±8	[30]
BPHMF	89	[31]

## 4 HMF as Modifier or Substituent in Conventional Condensation Adhesives

### 4.1 Wood adhesives

UF resins are thermosets that consist of linear and/or branched oligomers.^32^ Upon hardening, the UF resins form a 3D‐network that becomes insoluble and cannot be reshaped or melted. The high dry bond strength, a colorless glue line and a rapid curing, amongst other advantages, are the main reasons why UF resins have been extensively used as adhesives for wood‐based materials, and still are quite prominent today. Another major plus of UF resins is the low price of the raw materials, urea and formaldehyde. In comparison to the fossil‐based, cheap formaldehyde, renewables‐based HMF is a rather expensive raw material.

**Scheme 4:**
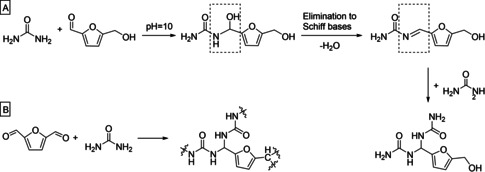
A) Reaction of urea and HMF proposed by Esmaeili et al.,^33^ involving possible formation of Schiff bases and aminals.^34^ B) Reaction of DFF and urea, as reported by Amarasekara et al.^35^

Esmaeili et al.^33^ synthesized HMF‐modified urea formaldehyde (UHMF) resin and compared it to genuine UF resin. They used the alkaline‐acid method, a procedure that involves pH changes, for the production of the UHMF and UF resin. First, the monomers – urea and paraformaldehyde or urea, paraformaldehyde and HMF – undergo methylolation under alkaline conditions (pH 10), after which the condensation of the UHF or UF resin is then concluded at pH 4. Esmaeili et al.^33^ also proposed the formation of the intermediate structure (R−CHOH−NH−C(O)−NH_2_) and its integration into the polymer chains after condensation. However, due to the high reactivity of the intermediate structure, it is very likely to eliminate water to form Schiff bases or undergo urea addition to give a 1 : 2 aminal‐type condensation product (Scheme 4A). Hui and Gandini^34^ showed that the polycondensation of furanoic dialdehydes with diamines led to the formation of polymeric Schiff bases. Amarasekara et al.^35^ have also proven this formation for diformylfuran–urea resins (Scheme 4B). They^33^ synthesized three UHF resins. The maximum amount of formaldehyde replacement was 29 mol.‐%. The viscosity, gelation time, solid content and water solubility were measured. They concluded that the higher steric hindrance near the carbonyl group in HMF lowered its reactivity compared to formaldehyde. In addition, the electrophilicity of the carbonyl group was decreased due to its conjugation with the – albeit weakly – aromatic furan ring structure. This led to a difference in the time needed for the polycondensation reaction with HMF compared to formaldehyde. The particleboards were pressed at 180 °C at a press factor of 55.4 s mm^−1^ and reached an IB strength of 0.55 N mm^−2^ which is significantly higher than the reference UF resin (0.39 N mm^−2^). Then again, a much lower press factor would have been sufficient for the UF resin. The density of the boards was not mentioned in the publication. Esmaeili et al.^33^ compared the results with those obtained from traditional UF synthesis and demonstrated the influence of HMF on the properties of a UF resin. A critical research question is whether it is possible to further optimize the synthesis in a way that more formaldehyde can be replaced, for example, by variation of synthesis parameters, such as pH, temperature or reaction time.

HMF has also been incorporated in melamine‐formaldehyde (MF) resins. MF resins are, for example, used for the impregnation of paper surface overlays. The higher water resistance and excellent weather resistance is the main characteristic that distinguishes MF resins from UF resins. However, compared to UF resins, MF resins are rather expensive. Cheap urea is added to produce melamine/urea/formaldehyde resins that can be used as exterior and semi‐exterior plywood and particleboards. Recently, Deng et al.^36^ substituted formaldehyde with glyoxal to decrease the formaldehyde emissions. They found that the melamine‐glyoxal (MG) resins have a higher activation energy than melamine/formaldehyde (MF) resins, shifting crosslinking to temperatures too high for particleboards. A melamine‐glyoxal resin modified with HMF was produced by Xi et al.^37^ for plywood testing. 5 % of an ionic liquid acid was used as hardener. Compared to MG resin, the HMFMG resin had a better bonding performance and had a dry shear strength of 1.03 N mm^−2^ with 80 % wood failure (MG=0.73 N mm^−2^). The soaking wet strength (24 h) was 0.73 N mm^−2^ with 65 % wood failure (MG=0.47 N mm^−2^). Wood failure is caused by adherend failure, not by adhesive failure. The results were compared to the requirements of the Chinese standard for plywood GB/T 9846 : 3‐2004 and fulfilled the minimum requirements of ≥0.7 N mm^−2^. The European standard for plywood EN 314–2 defines a shear strength between 0.2–0.4 N mm^−2^ and 60 % wood failure as minimum requirements for plywood classification 1 (testing after 24 h storage in cold water).

Extensive research was performed to determine the structures formed and to identify possible crosslinking bonds in the melamine‐glyoxal resin modified with HMF. Several possible structures were proposed based on analytical techniques, such as FTIR, CP‐MAS ^13^C NMR and MALDI‐TOF. The curing behavior was analyzed by DSC. Melamine‐glyoxal resin modified with HMF had a lower curing activation energy than the MG and was consequently more reactive. In addition, the curing of the melamine‐glyoxal resin modified with HMF took place at 108 °C, compared to the higher temperatures of 127 °C needed for curing MG resin. Thermomechanical analysis (TMA) found melamine‐glyoxal resin modified with HMF to have higher bonding strength, which was also confirmed by the mechanical testing of plywood shear strength. A previous study^38^ investigated the use of ionic liquids as hardener in urea‐glyoxal resins and found that curing was accelerated by the acidic part of the ionic liquid that was obtained by thermal hydrolysis during hot pressing. The stated advantages of ionic liquids as hardeners are that none of the two parts of the ionic liquid are volatile and after the pressing the almost neutral ionic liquid forms again, reducing the risk of hydrolytic damage to the wood surface. With regard to application in particleboard, the use of expensive ionic liquids as accelerators adds up to the high content of melamine and HMF, driving the price up. Apart from this, the high hydrophilicity and water solubility of ionic liquids might pose problems in applications.

AVALON Industries AG issued a patent^39^ that described the manufacturing process of thermally curable resins produced from a phenolic compound or an amino plastic forming agent with HMF. In 2017, they also launched a research project on the substitution of formaldehyde in UF and PF resins with HMF.^39^, ^40^ In the patent^39^ an example of full substitution of formaldehyde with HMF is given. The resins are produced at temperatures between 80–100 °C. In the given example, the urea/HMF resin was produced by stirring urea and 50 % HMF solution for 2.5 h at 90 °C at a pH of 2. For comparison, two samples were produced at 45 °C. The reaction was continued for several hours at 20 °C and the condensation checked by measuring the viscosity (*η*). Two types of urea‐HMF (UH) resins with a molar ratio of 1:0.5 (*η*=1275 mPa s) and 1:0.25 (*η*=65 mPa s) were synthesized and chipboard panels with 250 mm×250 mm×16 mm were produced. 10 % resin solid relative to the wood was applied and pressed at 220 °C. The internal bond strength was determined according to EN 319. The highest IB value was 0.55 N mm^−2^ obtained from a particleboard made with UH(1:0.5) with a density of 717 kg m^−3^ and a press factor of 28.1 s mm^−1^. A summary of the discussed HMF‐based wood adhesives is given in Table 2. An interesting research question in this context is whether the chemical structure of this resin compared to the resin that was produced in an alkaline‐acidic synthesis as proposed by Esmaeili et al.^33^ is different or similar.

**Table 2:** Mechanical properties of HMF‐based wood adhesives used in particleboard.

Press factor [s mm^−1^]	IB [N mm^−2^]	*T* [°C]	Density [kg m^−3^]	Resin [%]	Resin composition	Ref.
30.0	0.81	175			10 % sucrose, 0.3 % AlCl_3_, 2 % ethylene glycol	[19]
35.7	0.71	175			10 % sucrose, 0.5 % AlCl_3_, 1 % ethylene glycol	[19]
30.0	0.74	175			10 % sucrose, 0.6 % AlCl_3_, 0.25 % ethylene glycol	[19]
20.0	0.74	190			10 % sucrose, 0.67 % AlCl_3_, 3.3 % ethylene glycol	[19]
18.9	0.74	190			10 % sucrose, 0.6 % AlCl_3_, 2.5 % ethylene glycol	[19]
48.0	0.71	175			17 % sucrose, 0.4 % AlCl_3_, 0.9 % water, 1.75 % ethylene glycol	[19]
30.0	0.53	190			17 % sucrose, 1.2 % AlCl_3_, 1 % water	[19]
60.0	0.55	190			17 % sucrose, 0.3 % AlCl_3_, 1 % water	[19]
30.0	0.67	190			17 % sucrose, 0.6 % AlCl_3_, 1 % water	[19]
48.0	0.63	190			17 % sucrose, 0.2 % AlCl_3_, 0.45 % water, 10 % ethylene glycol	[19]
19.7	1.25	190			18.6 % sucrose, 0.4 % AlCl_3_, 1.78 % water, 1.62 % ethylene glycol, 2.36 % ammonium nitrate	[19]
21.8	0.05	180		10	100 % starch	[20]
21.8	0.06	180	685	10	90 % starch, 10 % sugar	[20]
21.8	0.13	180	750	10	80 % starch, 20 % sugar	[20]
21.8	0.08	180	754	10	70 % starch, 30 % sugar	[20]
21.8	0.25	180	760	10	80 % starch, 20 % sugar	[20]
21.8	0.40	108	759	10	53 % starch, 13 % sugar, 33 % tannin	[20]
66.7	1.40	220	800	30	tannin, sucrose, citric acid (25 : 75 : 50)	[21, 25]
66.7	1.45	220	800	30	tannin, sucrose, citric acid (25 : 75:0)	[21, 25]
25.0	0.02	220	553	12	citric acid, glucose, nitrogen source	[26]
45.0	0.37	220	598	12	citric acid, glucose, nitrogen source	[26]
50.0	0.46	220	565	12	citric acid, glucose, nitrogen source	[26]
50.0	0.46	220	617	12	citric acid, glucose, nitrogen source	[26]
50.0	0.44	220	592	12	citric acid, glucose, nitrogen source	[26]
50.0	0.43	220	591	12	citric acid, glucose, nitrogen source	[26]
50.0	0.41	220	610	12	citric acid, glucose, nitrogen source	[26]
50.0	0.46	220	615	12	citric acid, glucose, nitrogen source	[26]
50.0	0.40	220	613	12	citric acid, glucose, nitrogen source	[26]
50.0	0.47	220	630	12	citric acid, glucose, nitrogen source, wax	[26]
50.0	0.50	220	617	14	citric acid, glucose, nitrogen source, wax	[26]
25.0	0.05	220	638	12	citric acid, glucose, nitrogen source	[26]
45.0	0.25	220	627	12	citric acid, glucose, nitrogen source	[26]
50.0	0.35	220	567	12	citric acid, glucose, nitrogen source	[26]
20.6	0.27	220	733	10	urea, HMF (1:0.5)	[39, 40b]
20.6	0.21	220	729	10	urea, HMF (1:0.5)	[39, 40b]
28.1	0.55	220	717	10	urea, HMF (1:0,5)	[39, 40b]
28.1	0.52	220	718	10	urea, HMF (1:0,5)	[39, 40b]
20.6	0.43	220	715	10	urea, HMF (1:0,5)	[39, 40b]
20.6	0.43	220	718	10	urea, HMF (1:0,5)	[39, 40b]
28.1	0.44	220	714	10	urea, HMF (1:0.25)	[39, 40b]
24.4	0.39	220	715	10	urea, HMF (1:0.25)	[39, 40b]
20.6	0.31	220	712	10	urea, HMF (1:0.25)	[39, 40b]
28.1	0.36	220	713	10	urea, HMF (1:0.25)	[39, 40b]
55.4	0.55	180			urea, paraformaldehyde, HMF	[33]

### 4.2 Foundry industry

In the foundry industry, shaped metal parts with complex designs are often produced by using molds to fill the free volume. In the cold box process an aggregate, often sand, is coated with a curing binder and then blown into forms by air pressure. These molds are also referred to as core boxes. The choice of binder system is generally defined by the requirements of the specific molding process. In the application in sand molds, the strength of the sand mold, the moisture content and the permeability are important parameters. The strength of the sand mold has to be high enough to hold the shape under mechanical stress.

Battelle Memorial Institute^41^ described a cold‐box binder formulation with 5 wt% to 20 wt% HMF in an existing two‐component system comprising of two parts, phenol‐formaldehyde resin and isocyanate. The phenol‐formaldehyde resin had a molar ratio of F : P=1.3 : 1. The addition of HMF led to a reduction of the gel time of up to 38 % (86 s at 15 wt% HMF). The sand test tensile properties were measured after gassing, after 1 h bench life and after 3 h bench life. 5 wt% HMF replacement lead to no significant drop in the performance.

### 4.3 HMF‐glass fiber composites

An HMF‐based binder for mineral wool was described in a patent by Allais et al.^42^ The binder composition comprised HMF and ammonium, alkali metal or alkaline earth metal lignosulfonate. The thermoset is heat treated above 100 °C and applied to the rock or glass fibers by spraying. The intended application of this composites are insulation materials.

A commercial use of a possible HMF‐based binder is the ECOSE technology of Knauf Insulation GmbH for their glass wool insulation products.11a

## 5 Derivatives of HMF in Binder Systems

HMF is not only used directly but is also a key intermediate for valuable chemicals. Its derivatives are promising in adhesive production as well.

### 5.1 Bishydroxymethylfuran (BHMF)

Tang et al.^43^ performed a computational study on the incorporation of BHMF, furfural and furfuryl alcohol in a UF resin, concluding that the reactions of BHMF with urea are exothermic and thermodynamic favorable. BHMF has also been investigated in terms of resin production. Hu et al.^44^ produced a BHMF epoxy resin and compared this furan‐based epoxy resin to phenol‐based epoxy resins. There is also a patent from Golino^45^ that described mixing a BHMF resin with furfural and phenolic resins and studied the resulting binding forces between glass fibers. With the addition of BHMF to the resin, the flammability was reduced and the expansion rate was increased.^44^, ^45^, ^46^ BHMF has been used as a diol in the synthesis of polyurethane foams. Boufi et al.^47^ produced furan‐segmented polyurethanes. The thermoplastic elastomers, based on aromatic diisocyanate, BHMF and macrodiols as spacers, showed good mechanical and thermal performance.

**Scheme 5:**
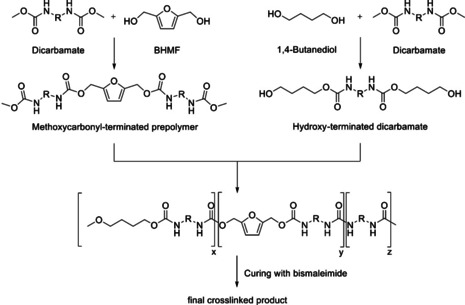
Production of non‐isocyanate polyurethanes (NIPUs) with BHMF as one component.

Zhang et al.^48^ studied the production of non‐isocyanate polyurethanes (NIPUs), for which purpose BHMF reacted with dicarbamates to produce methoxycarbonyl‐terminated prepolymers (Scheme 5).

Those prepolymers were then crosslinked with bismaleimide by means of a Diels‐Alder reaction with inverse electron demand. Zhang et al.^48^ named eco‐friendly PU products with recyclable, mendable and self‐healing properties as future applications of BHMF‐based NIPUS. No further investigation of the usage of adhesive were done for these systems

BHMF has also been used as starting material for the synthesis of 2,5‐bis[(2‐oxiranylmethoxy)methyl]‐furan (BOF) by reaction with epichlorohydrin. Hu et al.^49^ compared furan‐based epoxyamine thermosets (BOF) to 1,4‐bis[(2‐oxiranylmethoxy)methyl]‐benzene (BOB), that use bis(hydroxymethyl)benzene as starting compound. The authors investigated the curing behavior of the thermosets with DMA and analyzed the structure‐property relation with additional FTIR measurements. The influence of different curing agents was also investigated. BOF‐based epoxyamine thermosets were claimed to be a promising substitute for petroleum‐based thermosetting resins due to their higher glass transition temperature and higher storage modulus. However, the use of the toxic epichlorohydrin in BOF synthesis and the need of several reaction steps seem to be drawbacks that cannot easily be ignored.

### 5.2 Diformylfuran (DFF)

The Quaker Oats Company^50^ reported the utilization of DFF as co‐monomer in foundry binders comprising of furfuryl alcohol and an aromatic dialdehyde in 1982, only a rather small percentage of DFF (ca. 5 %), was used in the composition. Amarasekara et al.^35^ used DFF as a monomer for the synthesis of a DFF‐urea resin with a DFF/urea molar ratio of 1 : 4 (Scheme 4B). The product was a light brown, transparent, hard resin that was insoluble in water. Potential application fields were not addressed in the publication.

### 5.3 5‐Hydroxymethyl‐2‐vinylfuran (HMVF)

**Scheme 6:**
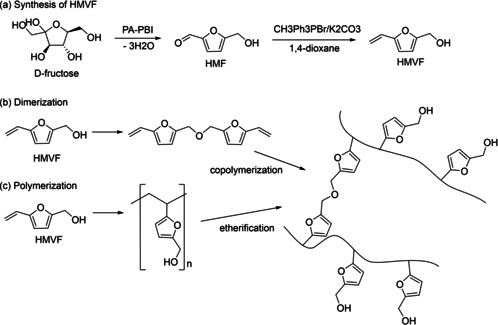
a) Dehydration of fructose to HMF by using phosphoric acid‐doped polybenzimidazole (PA‐PBI) and synthesis of HMVF (a).^52^ b, c) Curing of HMVF.^51^

Han et al.^51^ synthesized HMVF, as can be seen in Scheme 6, and investigated its potential as adhesive by testing its bonding ability to different surfaces, such as copper, aluminum, rubber and polyethylene. In general, the lab shear strength tests indicated that HMVF has affinity to polar substrate surfaces due to its hydroxy groups: steel (9.7±0.4 MPa)>copper (9.0±0.2 MPa)>aluminum (8.4±0.6 MPa)>PE (2.8±0.45 MPa)>rubber (0.38±0.01 MPa)>PTFE (0.31±0.02 MPa). The optimum curing conditions were at 110 °C and 2 h. Structural analysis suggested that the curing mechanism was driven by radical polymerization and additional crosslinking of HMVF. No solvents were needed for the curing reaction, rather the pure monomer was applied to the substrate surface where it underwent polymerization and crosslinking. The gradual buildup of the adhesion upon polymerization is analog to the curing mechanism of cyanoacrylates. HMVF showed similar lab shear strength on four tested surfaces (aluminum, copper, rubber, PE) compared to a conventional cyanoacrylate adhesive. HMVF was also compared in this study to conventional PVA and epoxy resins. It showed a better lab shear strength than those conventional resins on all tested surfaces. This indicates the enormous potential of an adhesive system based on HMVF. In addition, in vitro cytotoxicity was also investigated and showed HMVF to be innocent.

### 5.4 Furandicarboxylic acid (FDCA)

Gomes et al.^53^ patented a pressure‐sensitive adhesive made of furan compounds such as FDCA or its dialkyl esters, a second compound out of the group of a dimer of a fatty alcohol, as well as a crosslinking agent (isocyanate, epoxide, anhydride and maleimide). The adhesive was used to laminate a sheet or film.

The rich chemistry of FDCA in polycondensations towards replacements of terephthalic acid and polyterephthalates is not the subject of the present Review.

### 5.5 2,5‐Bis(hydroxymethyl)furan diacrylate (FDA)

**Scheme 7:**
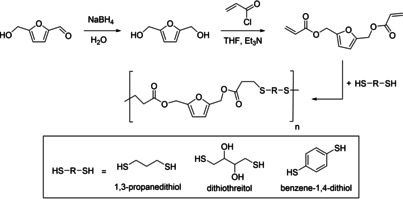
Synthesis of poly (β‐thioether esters) from 2,5‐bis(hydroxymethyl)furan diacrylate derived from HMF^54^ for the application as hot melt adhesive.^55^

2,5‐Bis(hydroxymethyl)furan diacrylate was synthesized from HMF.^54^ A thiol‐Michael addition was used as polymerization technique to produce poly(β‐thioether ester) derivatives of FDA (Scheme 7). Three dithiols, 1,3‐propanedithiol, dithiothreitol and benzene‐1,4‐dithiol, were used for the polymerization. Zhang and Dumont^55^ tested the application of polymers synthesized from dithiothreitol as hot melt adhesive due to the presence of hydroxy groups as side chains. In lab tests, wood substrates bonded with dithiothreitol‐based polymers exhibited an adhesive strength in lab shear joints of 1.5 MPa at room temperature. In comparison, a commercial hot melt resin for wood bonding, Stanley GS264BK, reached 2.1 MPa. In preparation and application, the strongly malodorous nature of mercaptans and thiophenols, already remarkably strong at very low concentrations, must be considered and might become a limiting factor.

## 6 Discussion of the Technological Performance of HMF‐Based Adhesives

### 6.1 Wood adhesives

**Figure 1:**
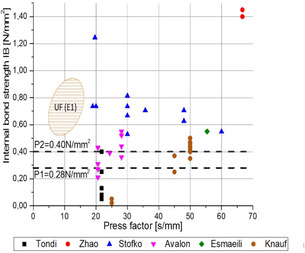
Internal bond strength of particleboard as a function of press factor for HMF‐based adhesives. The reference values according to EN 312 for 6–13 mm board thickness for P1 and P2 particleboards are indicated as dashed lines. Reference values for commercial UF resins are indicated as shaded field.11a Refernces: Tondi,^20^ Zhao,^21^, ^25^ Stofko,^19^ Avalon,^39^, 40b Esmaeili,^33^ Knauf.^26^

Many of the HMF‐based adhesive systems reported to date were tested in particleboard (Table 2). The internal bond (IB) strength is a main indicator for the strength. Figure 1 shows the IB strength as a function of the press factor. The press factor is a process‐related parameter, which is defined as the time needed to cure 1 mm of panel thickness. From an economic point of view, the press factor is a good indicator of processing costs, since the time needed for heating the panel during hot pressing and curing the adhesive is the main cost‐driving factor.11a Typical press factors for commercial adhesives depend on the adhesive system and the processing conditions. For an UF resin, typical pressing factors lie in the range of 5–12 s mm^−1^ on laboratory scale and 3–7 s mm^−1^ for industrial production at temperatures of 180–240 °C.11a As can be seen from Figure 1, none of the described HMF‐based adhesive systems come close to the target press factor. Currently, there is only one commercial, small‐scale HMF plant operating, with a capacity of 300 t per year.^14^ Consequently, the availability of HMF is still limited and needs to be significantly increased to render commercial production of HMF‐based adhesives economical. In situ HMF generation directly from carbohydrate feedstock is considered to be a promising alternative approach in binder production.

In general, particleboards are classified according to EN 312 into seven classes based on their properties. The standard particleboard (P2) is made for furniture manufacturing. The moisture content in the board should be in the range of 5–13 % (EN 322). Some of the particleboards produced by Avalon Industries^39^, 40b already reached the minimum requirements for P2 particleboard. In comparison with other more recently developed HMF‐based resins, the one patented by Avalon Industries showed one of the lowest press factors (20.6 s mm^−1^).

A closer look to the patent revealed that in a second step of the synthesis the resin was stirred at 20 °C for a time period of 4–168 h, during which a change in viscosity was observed. This raises the question of the storage stability of the produced resin. When comparing the results to those of Esmaeili et al.,^33^ it must be pointed out that by leaving out the alkaline step of a conventional UF synthesis a complete replacement of formaldehyde with HMF is possible. The obtained IB is comparable with the one from Esmaeili et al.^33^ It has to be noted that especially in comparison to the synthesis of UF resins, the mentioned reaction parameters (90 °C at pH 2) are rather harsh. Furthermore, the press temperature of the particleboard was much higher than those reported by Esmaeili et al.,^33^ who applied rather long press times of 12 min, which helped to reach higher temperatures in the core of the board.

The thermal characterization of their UHF resin indicated that the curing took place around 130 °C, compared to much lower temperatures of about 80 °C for a UF resin. These results highlight the importance of thermal analysis of resins for wood products. the highest IB values were reached by Zhao et al.^21^, ^25^ as can be seen in Figure 1. This was achieved by several factors. The rather high resin content of 20 wt% in combination with the high density of the boards (800 kg m^−3^) and the low thickness (9 mm) of the particleboard facilitated higher temperatures in the core of the board, which made it easier to reach higher IB values.

Typically, the press factor in the industrial production of particleboard is in the range of 3–5 s mm^−1^ at press temperatures of typically 200–240 °C, whereas, owing to the presence of water vapor, the temperature in the core of the particleboard does not exceed 110–120 °C. Using an untypically high press factor of 67 s mm^−1^ in combination with the high density and low thickness and smaller dimensions of the panels, the industrial core temperature limitation can be overcome on laboratory scale. Consequently, the described process is limited to the production of specialized, noncommercial particleboard. This makes a comparison with conventional products difficult.

A series of previous studies has indicated that curing of the HMF‐based adhesives takes place at temperatures above 120 °C, which is a major drawback for the production of particleboard. Although Xi et al.^37^ tested their HMFMG resin on plywood, which makes the comparison with particleboards inapplicable, the thermal analysis of this resin showed very promising results for the application in wood products in general. The thermal analysis of the HMFMG resins indicated a curing temperature of around 108 °C, which is much lower than the curing temperature of a MG resin at 127 °C. The results of the mechanical testing of the plywood boards showed fulfillment of the minimum requirements of the corresponding Chinese and European standards. Neither mechanical properties of the wood species itself, nor the density of the plywood panels were included in the publication, although both values influence the results of the mechanical testing. Consequently, the results should be treated with caution. An interesting topic for future research is the application of the HMFMG resin in particleboard, which would additionally enable a comparison with previous studies.

### 6.2 Composites

The application of HMF‐based binders in glass fiber composites has been reported. The application field of HMF‐based composites are wide, ranging from insulation materials to applications in aircraft or mass transit. For the binder production of the composites Zhao et al.^27^ used an in situ approach. The required material properties, such as tensile strength, will depend on the individual application.

In the manufacture of HMF‐based glass fiber composites, much higher manufacturing temperatures can be applied than in application of HMF‐based adhesives in wood materials. For the production of glass fiber‐PHMF composites Zhang et al. used curing temperatures as high as 180 °C, which is a clear advantage considering that thermal analysis of the PHMF showed the curing reaction to occur at around 150 °C.^29^ Zhang et al. used the tensile shear strength of long glass fiber reinforced thermosets made of PF resin as reference for their composites. Compared to this value (115 N mm^−2^), the composites reached rather satisfactory results (127 N mm^−2^). In general, there is no standardized reference tensile shear strength value for PF‐glass fiber composites as the needed strength strongly depends on the application. No specific application was mentioned in the publication by Zhang et al.^29^ which makes the comparison with commercial production difficult. Zhang et al. illuminated the potential applicability of PHMF in composites, whereas the application of this adhesive in other fields, such as in the wood industry, is rather problematic due to the high temperatures needed for curing.

## 7 Conclusion

This Review has evaluated the studies reported to date on adhesive systems based on HMF and its derivatives. In several cases, the technological performance in terms of internal bond (IB) strength of HMF‐based adhesives fulfilled the minimum requirements for particleboard. However, there are still some remaining issues that need to be solved before commercial application can be considered to be economically competitive. For example, this can be shown in the production of particleboard with HMF‐based adhesives. Many of the produced particleboards fulfilled the minimum IB strength that is required for P2 particleboard, but the press factor remains too high for an economic process. Overall, HMF‐based adhesives for particleboard do not yet reach the performance of commercial adhesives, such as UF.

The broad implication of the summarized findings is that the curing reaction is shifted to higher temperatures than for commercial adhesives. This has been proven by the thermal analysis of several HMF‐based resins. Although this outcome is less than optimal for the wood industry, it does not affect other application fields that are not restricted to lower temperatures during manufacture. This especially holds true for applications of HMF‐based adhesives in glass fiber composites and in the foundry industry.

With these results in mind, the number of reported adhesive systems based on HMF and its derivatives remains rather low, and the number of systems that can reasonably be expected to reach production is yet much smaller. Consequently, the optimization of the curing reaction of HMF‐based adhesives towards lower temperatures is a topic well worth investigating, in addition to new compositions with optimized reactivities. The range of reported application fields is wide and further research is more than justified. Taking the technological performance of the described HMF‐based adhesive systems into account, the applicability of HMF as a key reactant in adhesive systems is evident and clearly should be further explored in future research. As research into the applicability of HMF in adhesive systems is still in its infancy, further research is needed to produce economically viable and fully sustainable binder systems based on HMF and its derivatives, and this Review concludes with a plea to recognize the importance of scientific research on this topic.

## Conflict of interest

The authors declare no conflict of interest.

## Biographical Information

*Catherine Thoma received her master's degree in Material Science from Technical University Vienna in 2018. She now works as a junior researcher at Kompetenzzentrum Holz GmbH in the area of wood material technologies. She is pursuing a Ph.D. at BOKU University of Natural Resources and Life Sciences. Her research interests concern carbohydrate conversion and sustainable production of carbohydrate‐based resins for wood‐based panels*.